# Supplementary material for: Genetic Engineering of Klebsiella pneumoniae ATCC 25955 for Bioconjugate Vaccine Applications
Source: Microorganisms. 2023 May 17;11(5):1321. doi: 10.3390/microorganisms11051321 (PMC10222708; doi:10.3390/microorganisms11051321)
Supplement: Supplementary file 1 [file microorganisms-11-01321-s001.zip › microorganisms-2357089-supplementary-Figure S1.pdf]

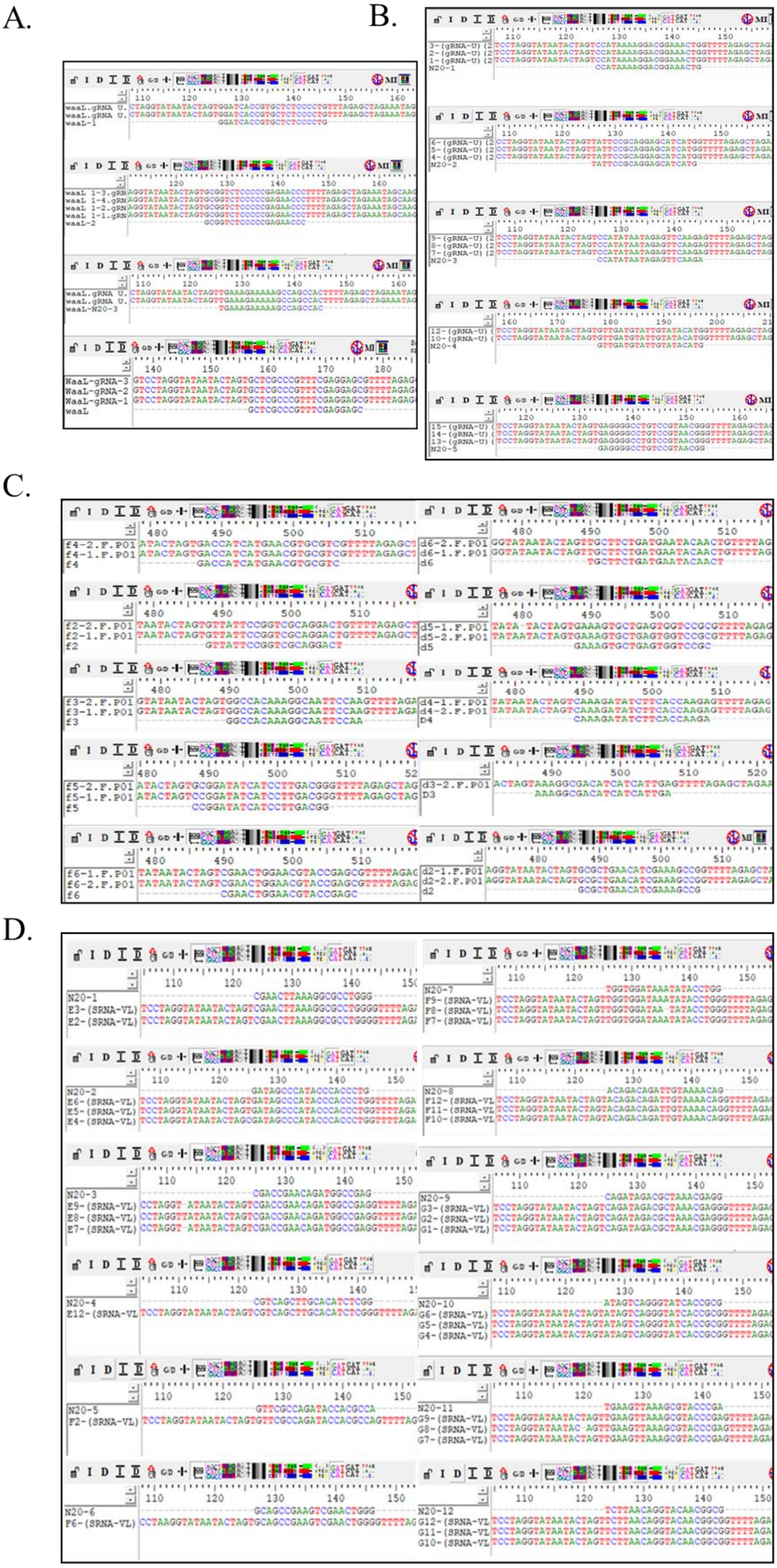
A.
B.
C.
D.

Supplemental Figure S1. Sequencing validation of each pTargetF-N20 plasmids.

(A) Validation of each N20 sites for *waaL* gene.

(B) Validation of each N20 sites for the wbbY-Z gene cluster.

(C) Validation of each N20 sites for the CPS gene cluster.

(D) Validation of each N20 sites for the ECA gene cluster.
